# Antibiotic use at a tertiary hospital in Tanzania: findings from a point prevalence survey

**DOI:** 10.1186/s13756-023-01317-w

**Published:** 2023-10-10

**Authors:** Denis Katyali, Godfrey Kawau, Bjørn Blomberg, Joel Manyahi

**Affiliations:** 1 Department of Microbiology and Immunology, Muhimbili University of Health and Allied Sciences, P.O. Box 65001, Dar es Salaam, Tanzania; 2 Department of Clinical Science, University of Bergen, Bergen, Norway; 3 National Centre for Tropical Infectious Diseases, Department of Medicine, Haukeland University Hospital, Bergen, Norway

## Abstract

**Background:**

In Tanzania, data on antibiotic use at the patient level is scarce, and intervention measures to optimize antibiotic use and reduce antimicrobial resistance are rarely performed.

**Objectives:**

To describe antibiotic use at Muhimbili National Hospital.

**Methods:**

This was a point prevalence survey on antibiotic use conducted at Muhimbili National Hospital in August-September 2022. The World Health Organization point prevalence survey data collection tool was used to collect patients’ information from the files. All patients admitted to the wards on the day of the survey were included.

**Results:**

Overall, 47% (185/397) of admitted patients were on at least one antibiotic during the survey. All antibiotics prescribed were for empirical treatment and guideline compliance was low, at 45%. Of 185 patients who received antibiotics, the most common indication was community acquired infection (55%) and 36% had no documentation of the reasons for prescribing antibiotics. Almost 75% of the antibiotics were administered parenterally, with only 2% switching to oral route. Microbiological tests were performed in only 9 (5%) patients out of 185 and results were available for only one patient. Of all participants, 52% received two or more antibiotic in combination, with the combination ceftriaxone-metronidazole being most frequently prescribed, followed by the combination of ampicillin, cloxacillin, and gentamicin. For individual antibiotics, ceftriaxone was the most frequently prescribed antibiotic accounting for 28% (79/283), followed by metronidazole (24%) and amoxicillin-clavulanic acid (11%).

**Conclusion:**

The findings of a high prevalence of antibiotic use, inadequate use of bacterial culture, and frequent empiric antibiotic treatment suggests the need for strengthening diagnostic and antimicrobial stewardship programs. Furthermore, this study has identified areas for quality improvement, including education programs focusing on prescription practice.

## Background

Antimicrobial resistance (AMR) in resource-limited countries is on the rise, with increasing number of infections caused by antimicrobial-resistant bacteria. The magnitude of AMR, its implications, and risk factors for its development are often not documented. Irrational use of antibiotics has been documented as driving the emergence of AMR. Despite this existing evidence, the use of antibiotics in resource-limited countries is poorly tracked, further fueling the emergency of AMR.

In Tanzania, studies have demonstrated a high burden of both community- and healthcare-acquired infections caused by antibiotic-resistant bacteria [1, 2]. Despite the known preventive measures for AMR, the burden is still on the rise. Previous studies from Tanzania have documented a high proportion of methicillin-resistant *S. aureus* and extended-spectrum beta-lactamase-producing bacteria causing surgical site infections and bloodstream infections.

 [3, 4], with these multi-drug-resistant bacteria predicting mortality and prolonged hospital stays [5–7].

The overall prevalence of antibiotic use in health care facilities in Tanzania has been reported to vary from 30 to 62%, depending on the level of the facilities [8–11]. There has been increased use of World Health Organization (WHO) “watch” category antibiotics in hospital settings in Tanzania [8]. Ceftriaxone, a third-generation cephalosporin, has been the most prescribed WHO “watch” category antibiotic at zonal, regional, and district hospitals [9]. WHO-not recommended antibiotics have been frequently used in Tanzania, despite WHO suggestions of a lack of evidence on the use of these antibiotics to treat infections. However, data on antibiotic consumption at the national level has shown increased consumption of both “access” and “watch” groups of antibiotics [12, 13]. But the use of these antibiotics is difficult to track as they are sold over the counter and consumed by humans, animals, and in aquaculture.

Misuse and overuse of antimicrobial agents are well known to drive the emergence of antimicrobial resistance through selective pressure. Monitoring antibiotic use by patients and antibiotic prescription behavior by health care workers is essential for planning containment measures to prevent the spread of AMR and optimize antibiotic use. Point prevalence surveys (PPS) on antibiotic use have been successful worldwide in optimizing antibiotic use in hospitals and planning interventions to reduce AMR [14–16]. It is also an important tool for monitoring quality improvement in health care. At Muhimbili National Hospital, Tanzania, like in many other low- and middle-income countries, data on antibiotic use at the patient level is scarce, and intervention measures to optimize antibiotic use and reduce AMR are rarely performed. Therefore, we conducted this study to understand the prevalence of inappropriate antibiotic use.

## Materials and methods

### Study setting

This study was conducted at Muhimbili National Hospital (MNH) for the duration of three consecutive weeks, starting from August 29, 2022, to September 18, 2022. MNH is located in Dar-es-Salaam, the economic hub of Tanzania. MNH has a 1500-bed capacity and is the largest tertiary and main consultant referral hospital in the country, attending approximately 2000 outpatients per day. MNH regularly receives referrals from other hospitals and has highly specialized staff, technical equipment, specialized imaging units, and a well-equipped clinical laboratory. It also serves as a teaching hospital for Muhimbili University of Health and Allied Sciences.

The hospital wards include pediatric wards (pediatric medical wards, pediatric surgical wards, pediatric oncology wards, and pediatric intensive care unit), neonatal wards (neonatal medical wards and neonatal intensive care unit), adult wards (adult medical wards, adult surgical wards, adult high-risk wards, adult intensive care unit, obstetric wards, and gynecology wards), and mixed wards (otorhinolaryngology and ophthalmology wards).

### Study procedures

This study adopted the WHO methodology for a PPS on antibiotic use in hospitals [17]. The study included all patients (irrespective of age and gender) admitted to MNH wards before 8:00 a.m. on the day of the survey. The sampling was done in each ward on the day of the survey by first preparing a list of all eligible patients according to the inclusion criteria. The list was ordered alphabetically according to patients’ surnames. We randomly selected between the first three patients on the list as the starting point for sampling, and from this list, every third patient was selected until the end of the list. In the event that a selected patient was not present in the ward, the next person on the list was selected. To minimize the impact of patients moving between wards, each ward was completely surveyed in one day. Each day, one ward was surveyed until the completion of all 39 wards. To maintain consistency in data collection, two research assistants with medical backgrounds were trained on the WHO PPS protocol for two weeks. Following the completion of the training, we piloted the tools for two days and reviewed the data generated to ensure data quality. Then, during data collection, approximately 15% of files were randomly double-checked by the lead investigator to ensure data quality.

Data were collected using a modified Excel WHO PPS data collection tool. The tools consisted of wards, patients, indications, and antibiotic information. All information was collected from the patient’s files or other medical records, including the hospital information management system. No patient interviews and no clinician or health facility staff interviews were performed.

### Data analysis

The collected data was analyzed using Stata version 16 (StataCorp LLC, College Station, Texas, USA). Categorical variables were presented as frequencies, percentages, and proportions. The chi-square test was used to compare the association between two categorical variables. A *p-*value of < 0.05 was considered statistically significant.

## Results

### Demographic and clinical characteristics

A total of 397 patients were surveyed between August 29, 2022, to September 18, 2022.

Participants aged 16–65 were most common (62%, 246/397), followed by children 1 month-15 years (16%, 63/397), neonates (12%, 47/397) and the elderly aged > 65 (10%, 41/397). 56% were females. Surgical (26%, 101/397), adult medical (22%, 87/397) and Obstetrics/gynecology (20%, 81/397) wards accounted for the highest number of patients. One-fourths and 72% (300/397) of patients, had urethral catheter and peripheral vascular catheterization, respectively. Table 1.

**Table 1:** Demographic and clinical characteristics of the study participants (n = 397)

Variables	Frequency (n)	Percentage (%)
**Age**		
< 1month	47	12
1month − 15 years	63	16
16–65 years	246	62
> 65	41	10
**Sex**		
Female	222	56
Male	175	44
**Wards**		
**Adult ICU**	16	4
Adult Medical ward	87	22
Adult surgical ward	101	26
Obstetrics/gynaecology ward	81	20
Pediatric medical ward	65	16
Neonatal ward	47	12
**Urethra catheterization**		
No	300	76
Yes	97	24
**Peripheral vascular catheterization**		
No	112	28
Yes	285	72
**Intubation**		
No	376	95
Yes	21	5

### Prevalence of antibiotic use

Overall, 185/397 (47%) of admitted patients received one or two antibiotics during the survey. There were no differences in antibiotic use by sex, age groups, intubation or type of wards where patients were admitted. Patients with urethral catheter (64%, 62/97) were more frequently on antibiotic treatment compared to those who were not catheterized (41%,143/300), p-value < 0.001 (Table 2). Participants with vascular catheters were more frequently on antibiotic treatment (61%, 173/285) compared to those without (11%, 12/112), p value < 0.001.

**Table 2:** Prevalence of antibiotic use among participants (n = 397)

Variables	Total (n)	Antibiotic use		p-value
		Yes, n (%)	No, n(%)	
**Overall**	397	185 (46)	212(54)	
**Age**				
< 1month	47	27(57)	20(43)	reference
1month − 15 years	63	29(46)	34(54)	0.24
16–65 years	246	107(43)	139(57)	0.05
> 65	41	22 (46)	19(54)	0.73
**Sex**				
Female	222	94(42)	128(58)	
Male	175	91 (52)	84(48)	0.05
**Wards**				
**Adult ICU**	16	7(44)	9(56)	reference
Adult medical ward	87	47(54)	40(46)	0.45
Adult surgical ward	101	53(52)	48(48)	0.52
Obstetrics/gynaecology ward	81	21(26)	60(74)	0.16
Pediatric medical ward	65	30(46)	35(54)	0.86
Neonatal ward	47	27(57)	20(43)	0.34
**Urethra Catheterization**				
No	300	143(41)	177(59)	
Yes	97	62(64)	35(36)	< 0.001
**Peripheral vascular catheterization**				
No	112	12(11)	100(89)	
Yes	285	173(61)	112(39)	< 0.001
**Intubation**				
No	376	174(46)	202(54)	
Yes	21	11(52)	10(48)	0.59

### Patterns of antibiotics use and indications

All antibiotics prescribed were for empirical treatment and guideline compliance was low at 45%. Of 185 patients who received antibiotics, 101 (55%) had community acquired infection as indication, 67 (36%) were given antibiotics without documentation of the reasons for prescribing antibiotics, and for 11 (6%) patients the indication was hospital associated infection. The most common clinical diagnoses for patients who received antibiotics were respiratory tract infections (15%, 28/185), gastrointestinal tract infections (15%, 28/185) and genital or urinary tract infections (14, 27/185). However, a disproportionate number of patients received antibiotics without documentation of the clinical diagnosis (37%, 67/185). For the majority of the patients antibiotics were dosed twice (29%, 53/185) or thrice daily (31%, 58/185). Across all patients, almost 75% of antibiotics were administered parenterally, with only 2% switching to oral route. Of all, 43% received a combination of two antibiotics and 9% received more than two antibiotics. Of participants receiving two or more antibiotics, the combination of ceftriaxone and metronidazole was the most common (24% of all prescribed antibiotics), followed by ampicillin, cloxacillin, and gentamicin (6% of all prescribed antibiotics).

Culture sample were performed in only 9 (5%) patients out of 185 and results were available for only one patient (Table 3).

**Table 3:** Patterns of antibiotic use among participants (N = 185)

Variables	Frequency (N)	Percentage (%)
**Number of doses per day**		
One	74	40
Two	53	29
Three	58	31
**Route of antibiotics administration**		
Oral	47	25
Parental	138	75
**Switch to oral administration**		
No	135	98
Yes	3	2
**Type of treatment**		
Empiric	185	100
Directed treatment	0	0
**Guideline compliance**		
Yes	84	45
No	101	55
**Patient on antibiotic combination**		
One	89	48
Two	79	43
Three or four	17	9
**Indication type**		
Community acquired infection	101	55
Hospital acquired infection	11	6
Not documented	67	36
Surgical prophylaxis	6	3
**Clinical diagnosis**		
Respiratory tract infections	28	15
Gastrointestinal tract infections	28	15
Genital-urinary tract infections	27	15
CNS-bloodstream infections	18	10
Borne and soft-tissue infections	17	9
Unknown	67	36
**Culture sample taken**		
No	176	95
Yes	9	5

### Antibiotic prescription

**Fig. 1:**
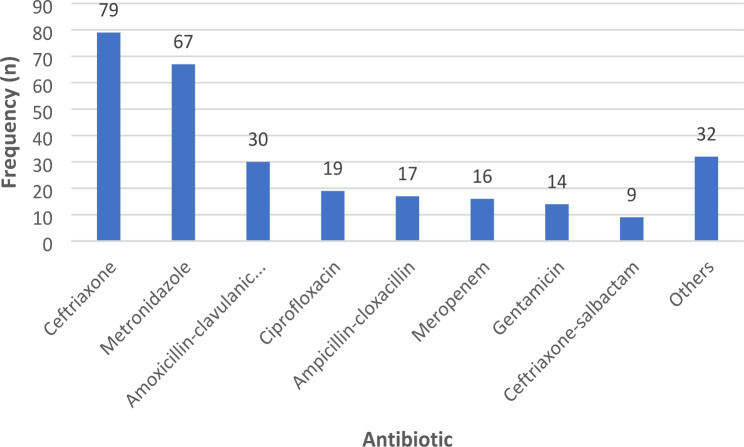
Frequency (n) of the antibiotic use among the participants

A total of 283 antibiotic prescriptions were made during the survey. The most prescribed antibiotic during the survey period was third generation cephalosporins (ceftriaxone) accounting for 28% (79/283), followed by metronidazole 24% (67/283), amoxicillin-clavulanic acid 11% (30/283) and ciprofloxacin 7% (19/283). Figure 1. Ampicillin-cloxacillin and ceftriaxone-sulbactam, which are not WHO-recommended antibiotics, were prescribed in 6% (17/283) and 3% (9/283) of prescriptions, respectively.

## Discussion

Tanzania is currently implementing its national action plan 2023–2028 to combat antimicrobial resistance, and the use of PPS on antimicrobial use to improve antibiotic prescription is one of the core activities in strategic objective number three [18]. We conducted this PPS at a tertiary hospital in Tanzania and found the overall prevalence of antibiotic use to be 47%, which was higher than recommended by WHO, 40% [17]. However, studies from tertiary facilities in low- and middle-income countries have reported varying prevalence of antibiotic use. A recent study in three tertiary-level facilities in Kenya reported the prevalence of antibiotic use at 46%, comparable to in our study [19]. In an earlier study at a tertiary hospital in Ghana, the prevalence of antibiotic use among inpatients was 51% [20], higher than reported in our findings and Kenya.

In this survey, antibiotic prescriptions were entirely empiric without evidence from microbiological test results, which could have illuminated the causative bacteria and effective antibiotics. Bacterial culture in this survey was only requested for 5% of the participants. While this is in line with other studies from the region [9], it is disheartening that almost all patients are treated blindly by clinicians with no evidence of the causative pathogens. The underutilization of microbiology services suggests that either clinicians have little trust in the microbiological tests or they have little knowledge of the importance of culture and susceptibility in guiding treatment. On the other hand, concern about the burden of cost shared by the patient for microbiological tests may lead clinicians to avoid requesting culture to save patients from added expenses. In other circumstances microbiological testing was requested by clinicians but never performed, as patients had to pay first before investigations are done. This leads to the conclusion that addressing AMR not only requires knowledge but also a focus on structural dimensions like poverty and a lack of national health insurance. Antibiotics in this setting are regarded as quick fixes for care, productivity, hygiene, and inequality [21]. Regardless, the result is extensive empiric prescriptions with likely overuse and misuse of antibiotics. Furthermore, patients are denied of the potential benefits of microbiological results in terms of receiving more targeted, narrow-spectrum and more affordable treatment. These findings highlight an obvious opportunity for quality improvement projects on diagnostic and antimicrobial stewardship programs at this tertiary facility. The overall prevalence of antibiotic use was within the estimated prevalence from other studies performed in Tanzania [8, 10, 11].

We observed that ceftriaxone, which belongs to the WHO “watch” category, was the most commonly prescribed antibiotic, followed by metronidazole and amoxicillin-clavulanic acid, both belonging to the WHO “access” group of antibiotics. However, ampicillin-cloxacillin and ceftriaxone-sulbactam, which the WHO does not recommend for use in clinical practice, were prescribed to some patients. Prior studies in Tanzania showed similar patterns of antibiotic prescriptions, where ceftriaxone, metronidazole, and amoxicillin-clavulanic acid were frequently prescribed antibiotics [8, 9]. Furthermore, studies from Sub-Saharan Africa have reported a similar trend in the use of antibiotics with extensive use of ceftriaxone [8, 9, 19, 22, 23], the notion of ceftriaxone being broad-spectrum and easy to use as a single dose per day remains a reason for its increased usage. Increasing resistance to ceftriaxone in Gram-negative patients has been reported at the same health facility [5, 24, 25], and extended-spectrum beta-lactamase-producing Gram-negatives have been reported in up to 50% of isolates in Tanzania [26–28]. Therefore, the effectiveness of ceftriaxone as a first-choice empiric antibiotic needs to be revisited at this facility, as this antibiotic has a relatively high risk of selection for bacterial resistance. In addition, the observed pattern of prescription calls for the need for this facility to create an annual cumulative antibiogram, which could help clinicians and pharmacists make appropriate choices for empiric antibiotic treatment.

In this survey, we found that parental routes of antibiotic administration were common, and that only 2% of those switched to oral routes of administration during the whole course of hospital admission. These findings indicate that in ward rounds, antibiotic prescriptions are not discussed in detail, and decisions about the escalation or de-escalation of antibiotics are certainly not often considered. Obviously, the lack of microbiological results makes it even more difficult to target and de-escalate antibiotic treatment. Similar findings have been reported in other studies from the country and other developing countries [19, 22, 29]. The findings call for the establishment of a dedicated antimicrobial stewardship committee, which should comprise microbiologists, pharmacists, and infectious disease specialists. The team should participate in ward rounds to provide advice and guidance on the prescription of antibiotics.

We observed that community-acquired infections were the most common indication for antibiotic administration, and one-third of patients received antibiotics without documentation of the reasons for prescribing antibiotics. In addition, compliance to the treatment guideline was low, which reflects that antimicrobial stewardship at this setting is rarely implemented. Despite community-acquired infections being a common indication, neither of these patients were confirmed to have bacterial infections as bacterial cultures were not performed. The consequence of this empiric treatment in a setting without a hospital cumulative antibiogram is the emergence and spread of antimicrobial resistance. Other studies from the region have documented the same findings, were community acquired infections are a common indication and limited documentation exists for the reason for antibiotic prescription [9, 19].

This study observed that 52% of patients received two (43%) or more (9%) antibiotics in combinations; ceftriaxone combined with metronidazole, followed by the combination of ampicillin, cloxacillin, and gentamicin were the most common. The combinations of ceftriaxone and metronidazole was likely targeting suspected polymicrobial infections (aerobic and anaerobic infections). The combination of ampicillin, cloxacillin and gentamicin may have been chosen to widen the coverage compared to individual drugs alone, reflecting that the clinicians treat blindly without any microbiological evidence to guide therapy. Often, the use of combination antibiotics in empirical therapy is considered to widen the coverage of susceptibility over individual antibiotics alone. This means the second antibiotic offers increased coverage compared to the first antibiotic. The practice of combining metronidazole with other antibiotics without considering the presence or absence of risk factors for anaerobic infections has been common in sub-Saharan Africa [9, 20, 22, 23, 30]. It is unfortunate that culture and susceptibility testing for anaerobic bacteria are rarely performed, and the bacterial susceptibility pattern to anaerobes is not known.

A weakness of this study is that it is a single-center report, and the findings may not reflect practice in other settings. However, a strength of the study is that by assessing a main national and teaching hospital in Tanzania, the study is well situated to highlight gaps in knowledge and practices on addressing AMR and provide opportunities for improvement in combating AMR. Another strength of the study is that it addresses a topic that has been under-researched across the continent. Despite being a single-center study, our findings are similar to those studies from sub-Saharan Africa, [8–10, 19, 20, 29, 30], where AMR is endemic and associated with increased morbidity and mortality.

In conclusion, the findings of a high prevalence of antibiotic use, underutilization of bacterial culture, and total reliance on empiric antibiotic treatment suggest the need for strengthening diagnostic and antimicrobial stewardship programs. The management should provide the committee with resources, including human resources like microbiologists and infectious diseases specialists who should be dedicated to performing bedside consultations for antimicrobial use, PPS, and prospective audits on antimicrobial use and providing feedback. Furthermore, this study has identified areas for quality improvement, including education programs for healthcare workers on antibiotic prescription behaviors and practice change, as well as training of healthcare workers on the importance of performing culture and susceptibility.

## Data Availability

The datasets used and/or analyzed during the current study are available from the corresponding author upon reasonable request.

## List of Abbreviations

AMR: Antimicrobial resistance
MNH: Muhimbili National Hospital
WHO: World Health organization
